# Spatial Heterogeneity Influences of Environmental Control and Informal Regulation on Air Pollutant Emissions in China

**DOI:** 10.3390/ijerph17134857

**Published:** 2020-07-06

**Authors:** Zhenhua Zhang, Guoxing Zhang, Shunfeng Song, Bin Su

**Affiliations:** 1 School of Management, Lanzhou University, Lanzhou 730000, China; 2 Institute of Green Finance, Lanzhou University, Lanzhou 730000, China; 3 College of Business, University of Nevada Reno, Reno, NV 89557, USA; song@unr.edu; 4 Energy Studies Institute, National University of Singapore, Singapore 119620, Singapore; subin@nus.edu.sg

**Keywords:** air pollution control policy, informal environmental regulation, spatial econometric model, policy quantification, China

## Abstract

High air pollutant emissions in China have become serious environmental issues threatening public health. While spatial heterogeneity plays an important role in environmental regulation in China, it is necessary to analyze the spatial heterogeneity influences of air pollution control policies and informal environmental regulation on air pollutant emissions in China. Based on the quantification of air pollution control policies, this paper incorporates the central government’s policy formulation and local government’s policy implementation into the intensity of air pollution control policy. This paper uses the panel data of China’s 30 provinces to examine the spatial impact of air pollution control policy and informal environmental regulation on air pollutant emissions. The results show that (a) air pollutant emissions represented by soot and dust emission intensity has a significant positive spatial spillover effect; (b) air pollution control policy and informal environmental regulation play significant inhibitory roles in air pollutant emissions; (c) informal environmental regulation has a negative moderating effect on the negative relationship between air pollution control policy and air pollutant emissions. Other implications for environmental management have also been discussed.

## 1. Introduction

Since the reform and opening-up in 1978, China has experienced significant economic growth, rapid industrialization, and urbanization. Significant achievements in economic development have made China the single largest contributor to global sulfur dioxide (SO_2_) emissions and have generated significant amounts of soot and dust (SD) emissions. China’s air pollutant emissions are the worst in the world if it is measured by the environmental concentration of SO_2_ and particulate matter (PM) [1]. High SO_2_ emission levels have led to the formation of acid rain, which has caused considerable damage to the ecosystem. High SD emissions are associated with a significant increase in PM_2.5_ and increase the risk of cardiovascular and cerebrovascular disease [2]. In the face of air pollutant emissions and its hazards, a real problem that needs to be solved is as follows: how can China’s various environmental regulations effectively control air pollutant emissions?

With the implementation of the sustainable development strategy, China has gradually strengthened pollution control and environmental protection. To date, China has established a multi-participatory air pollution governance system, including air pollution control policy and informal environmental regulation. Air pollution control policies are primarily designed by the central government but are implemented by local governments [3]. In addition, as Chinese citizens’ awareness of environmental protection has increased, public actors such as ordinary citizens and news media have also participated in the process of environmental governance, forming informal environmental regulation [4].

Spatial heterogeneity plays an important role in environmental regulation in China. It has been noted that China’s air pollution patterns have obvious spatial characteristics [5]. To date, most empirical studies have analyzed the impact of air pollution control policy on pollution [6], and the impact of informal environmental regulation on pollution [7]. However, more empirical evidence is needed to further support the spatial dependence of China’s air pollutant emissions. Most studies have focused on the implementation of air pollution control policy, ignoring the policy formulation factors of air pollution control policy in China [3]. Few studies investigate the possible moderating effect of informal environmental regulation on the relationship between air pollution control policy and air pollutant emissions [6,7].

The main contributions of this paper include the following: (a) we characterize the formulation intensity of air pollution control policy based on the content of policy literature. Policy formulation factors of the central government and the policy implementation factors of local governments are included in the process of constructing the intensity of air pollution control policy. (b) We reflect the spatial characteristics of air pollutant emissions in details in various regions of China. Using spatial econometric models can avoid estimation bias due to ignoring the spatial correlation of provincial air pollutant emissions. (c) Considering the spatial dependence of provincial air pollutant emissions, we study the impact of air pollution control policy on air pollutant emissions in China’s context and investigate the moderating effect of informal environmental regulation on the relationship between air pollution control policy and air pollutant emissions systematically. The results provide new insights into the control of atmospheric pollutant emissions in various provinces in China.

The rest of this paper is organized as follows. Section 2 provides a literature review. Section 3 introduces research design. Section 4 analyzes the variables and data. Section 5 gives the estimation results of the empirical model and gives the results of the robustness test. Section 6 summarizes the main findings and proposes policy recommendations.

## 2. Literature Review

Collaborative Governance Regime emphasizes the design and implementation of environmental regulation by formal decision-makers and informal public actors [8]. According to the existing literature, air pollution control is contributed by air pollution control policy and informal environmental regulation [4,6,7,9,10]. This section reviews the studies analyzing the impacts of air pollution control policy, and informal environmental regulation on air pollutant emissions.

### 2.1. Air Pollution Control Policy

Air pollution control policy refers to policy instruments such as command-and-control instruments (such as emission standards) and market-oriented instruments (such as environmental taxes) implemented by public authorities to control atmospheric pollutant emissions [4]. Due to the severe health damage caused by air pollution, air pollution control policy was first adopted by developed countries since the 1950s, such as the UK Clean Air Act of 1956 and the Clean Air Act in the US, originally passed in 1963 and amended in 1970, 1977, and 1990 [11]. With the acceleration of industrialization, air pollution control policies have been applied in many developing countries [12] such as China, India, and Mexico.

Most studies have found a positive correlation between air pollution control policy and environmental performance. Some studies explored the positive effects of specific policies on reducing air pollutant emissions from the field of fuel policy, vehicle restriction policy, and corporate emissions policy. For example, in terms of fuel policy, Auffhammer and Kellogg [9] used a Difference-in-Differences model based on panel data to conclude that due to clear regulatory objectives, the California gasoline content standard significantly decreased ozone concentrations by 7% and 10% in both urban and suburban areas. In terms of traffic control, Viard and Fu [10] found that Beijing’s vehicle restriction policy improved air quality to some extent through a piecewise regression model, and air pollution fell 21% during one-day-per-week restrictions. In terms of corporate emissions, using industry-level data on bilateral trade between China and 14 EU countries, Marconi [6] found that environmental regulation policies have significantly reduced corporate emissions. Other studies discussed air pollution control effects from specific policy perspectives such as economic regulation policies [13], automotive smoke inspection policies [14], and sulfur control regulation [15]. There are also studies raising the opposite views. For example, by investigating SO_2_ emissions from the US coal industry, Schlottmann [16] concluded that there is no link between air pollution control policy and atmospheric pollutant emissions. Blackman and Kildegaard [17] studied the inspections conducted by an environmental agency at a Mexican tannery, and showed that pollution reduction has nothing to do with the implementation of clean technology.

In the literature, some indicators have been used to measure the intensity of air pollution control policy, such as sewage charges [18] and input cost of pollution reduction [19]. However, these studies focus on proxy variables of air pollution control policy in different regions, which may lead to different conclusions in the same country or region using different proxy variable indicators and affect the accuracy of policy effect evaluation. Moreover, existing research has paid more attention to the factors of policy implementation, and less attention has been paid to the formulation of air pollution control policy based on the content of the policy text. Based on the existing policy implementation level, construction of the formulation intensity of air pollution control policy using quantitative indicators at the policy formulation level can reflect the efforts of different levels of government to control air pollutant emissions truly [20].

### 2.2. Informal Environmental Regulation

Informal environmental regulation refers to actions taken by citizens and relevant citizen groups to change the behavior of polluting companies [21,22]. Normally, these actions include citizen resistance to company products, environmental requirements from citizens to the government, and media coverage of environmental cases. Informal environmental regulation is often seen as a powerful complement to air pollution control policy. Regarding the impact of informal environmental regulation on pollution control, the existing literature has two opposing views. Some studies demonstrated the positive role of informal environmental regulation in reducing pollutant emissions from the perspective of media environmental supervision in India [7], or environmental Non-Governmental Organizations (NGOs) in China [4]. Contrary to the above findings, some scholars found no evidence to prove that there is an important relationship between pollutant emissions and China’s informal environmental regulation [23]. Therefore, facing these disputes, it is necessary to further study the role of China’s informal environmental regulation in reducing air pollutant emissions.

Informal environmental regulation may play a moderating role in the relationship between air pollution control policy and air pollutant emissions. When air pollution control policy is weak or absent, informal environmental regulation not only complements air pollution control policy, but also provides feedback to improve the design and implementation of air pollution control policy [24]. As companies may be concerned that their products are resisted, and local governments may be concerned about media disclosure of environmental issues, they must comply with environmental standards set by local communities or environmental organizations [21]. Moreover, in areas where civil society is underdeveloped, the public may also exert pressure on local governments to implement air pollution control policy [25]. Public participation in environmental monitoring can reduce the likelihood of non-compliance incidents and increase the efficiency of environmental regulation [26]. Some scholars demonstrated that corporate environmental performance is influenced by both formal and informal regulation and that formal regulation is largely influenced by informal regulation [22]. Informal environmental regulation may take effect when there is a gap between air pollution control policy and local environmental preference, or when informal environmental regulation can impose politics, society, or economy pressure.

### 2.3. Comments

The spatial relationship is a known phenomenon in ecological research and refers to the relationship between certain variables observed in different regions [5]. As environmental pollution has spatial spillover effects, air pollution in one region is not independent of other regions. In addition, China’s various regions are closely linked, and the environmental pollution caused by economic development and industrial layout is also widely connected. The closer the provinces are, the closer these connections are. If ignoring the effects of the possible spatial dependence, the model estimates would produce significant biases or false verification. Moreover, these studies considering spatial characteristics ignore the moderating effect of informal environmental regulation on the relationship between air pollution control policy and air pollutant emissions, which is not conducive to exploring the heterogeneity of the spatial impact of air pollution control policy on air pollutant emissions. Therefore, it is of great practical significance to explore the spatial impact of air pollution control policy and informal environmental regulation on air pollutant emissions, especially the moderating effect that informal environmental regulation may play in the relationship between air pollution control policy and air pollutant emissions.

## 3. Research Design

**Figure 1:**
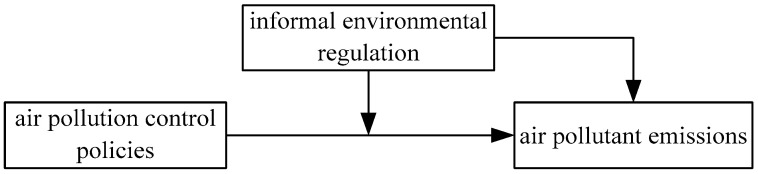
Conceptual relationship among air pollution control policies, informal environmental regulation, and air pollutant emissions.

Collaborative Governance Regime emphasizes the important roles of formal decision-makers and informal public actors in the process of environmental regulation [8]. The current Chinese air pollution governance system includes air pollution control policy and informal environmental regulation implemented on polluting industries or behaviors by various participants [7]. Governments at all levels, including the central government and local authorities, are primarily responsible for the design and implementation of air pollution control policy [3]. Public actors such as public citizens and news media promote air pollution governance through informal environmental regulation [4]. The informal environmental regulation driven by public actors greatly complements air pollution control policy driven by governments at all levels in the aspects of controlling air pollutant emissions [7]. Figure 1 shows the main conceptual relationship among air pollution control policy, informal environmental regulation, and air pollutant emissions according to the existing literature [3,4,7,8].

### 3.1. Non-Spatial Econometric Model

The IPAT equation was first proposed by Ehrlich and Holdren [27], which was a classic equation for assessing the effect of human activities on environmental change. After that, Dietz and Rosa [28] put forward STIRPAT model by putting IPAT into a stochastic model, which can statistically stimulate the non-proportionate impacts of different variables on environmental change. The STIRPAT model is the basic theoretical framework for the study of environmental pollution influencing factors [29]. It is in the form of $I_{it}=aP_{it}^{b}A_{it}^{c}T_{it}^{d}e_{it}$, where *I*, *P*, *A*, and *T* represent environmental impact, population size, per capita wealth, and science and technology (S&T), respectively. The major advantage of the STIRPAT model is that it allows coefficients to be estimated as parameters, and appropriate decomposition and improvement of various impact factors.

The socioecological theory put forward the requirements that the relationship hypotheses between anthropogenic factors and environmental impacts should not simply be assumed within the model structure, which means it should be testable by empirical evidence [30]. Therefore, we use the modified STIRPAT model considering air pollution control policy and informal environmental regulation as the explanatory variables to illustrate the conceptual relationship in Figure 1. We explore the impact of air pollution control policy and informal environmental regulation on air pollutant emissions in China by applying the modified STIRPAT model, without considering the spatial correlation between adjacent provinces.

(1) $$\ln Y_{it}=\alpha_{i}+\beta_{1}\ln PSPE_{i,t}+\beta_{2}\ln PPTC_{i,t}+\beta_{3}\ln PSPE_{i,t}\times \ln PPTC_{i,t}\;+\beta_{4}\ln SEE_{i,t}+\beta_{5}\ln PSPE_{i,t}\times \ln SEE_{i,t}+\sum_{k=1}^{h}\gamma_{k}\ln C_{ik,t}+\varepsilon_{it}$$

where all variables in Equation (1) are natural logarithms. *i* (*i* = 1, …, *N*) represents the province and *t* (*t* = 1, …, *T*) represents time; γ indicates the total number of control variables. $Y_{it}^{}$ is the dependent variable, representing the air pollutant emissions expressed by the pollution intensity of SO_2_ and SD. α indicates the constant term. *PSPE*, *PPTC*, and *SEE* indicate the intensity of air pollution control policies, the public environmental participation, and the media environmental monitoring, respectively. $C_{ik,t}$ is a collection of the control variables mainly expressed from STIRPAT model, including economic development [31], industrial structure [5], S&T expenditure [32], energy consumption [33], population density [31], and the degree of opening-up reflected by foreign direct investment (FDI) [34]. The details of the variables can be seen from Section 4 below. $\varepsilon_{it}^{}$ is a random disturbance term, which indicates the unobserved error.

### 3.2. Spatial Estimation Method

Pollution in one place may be affected by neighboring areas and lead to spatial autocorrelation of space and time, resulting in the invalid ordinary least squares (OLS) estimates. Before establishing a spatial econometric model, we first test the existence and forms of spatial effects. In this paper, two Lagrange multiplier (LM) tests and other robust LM tests based on residuals obtained from panel regression are applied to perform non-spatial OLS model regression and diagnose error or lag space dependence.

We examine the spatial impact of air pollution control policy and informal environmental regulation on air pollutant emissions in China by considering spatial correlation. According to [35], three econometric models can be used to test the spatial correlation. The spatial autoregressive (SAR) model is the first model. The dependent variable’s value of region *i* is influenced by the adjacent dependent variable. In other words, air pollutant emissions in the region of *i* is affected by air pollutant emissions from neighboring regions. The SAR model is defined as:

(2) $$\ln Y_{it}=\rho \sum_{j=1}^{n}W_{ij}\ln Y_{jt}+\sum_{k=1}^{f}\beta_{k}\ln X_{ik,t}+\mu_{it}+\lambda_{it}+\varepsilon_{it}$$

where *f* indicates the total number of independent variables and control variables. $X_{ik,t}$ is a collection of the independent variables and control variables. ρ is the spatial parameter that reflects the spatial correlation of the sample observations. Therefore, it assesses the impact of atmospheric pollution emissions on local air pollutant emissions in adjacent areas. $\sum_{j=1}^{N}W_{ij}$ is a spatial weight matrix based on geographic distance; $W_{ij}$ is one element in the (*N* × *N*) spatial weight matrix; *N* represents the number of provinces. $\mu_{it}$ indicates the time fixed effect of the spatial unit. $\lambda_{it}$ indicates a spatial fixed effect.

Spatial error model (SEM) is the second model. It assumes that the spatial dependence is due to the error term from the dependent variable of the adjacent region. SEM is defined as:

(3) $$\ln Y_{it}=\alpha +\sum_{k=1}^{f}\beta_{k}\ln X_{ik,t}+\mu_{it}+\lambda_{it}+\varphi_{it}$$

(4) $$\varphi_{it}=\delta \sum_{j=1}^{n}W_{ij}\varphi_{jt}+\varepsilon_{it}$$

where $\varphi_{it}$ is the spatial dependence error term. δ is a spatial autoregressive coefficient that indicates the effect of residuals in adjacent regions on the residuals of local regions. $\varepsilon_{it}$ is the residual of independent and identically distributed (i.i.d).

Spatial Durbin model (SDM) is the third model, which assumes that the dependent variable in region *i* is dependent on independent variables and dependent variables spatially in some other adjacent regions. SDM is defined as:

(5) $$\ln Y_{it}=\rho \sum_{j=1}^{n}W_{ij}\ln Y_{jt}+\sum_{k=1}^{f}\beta_{k}\ln X_{ik,t}+\sum_{k=1}^{f}\theta_{k}\sum_{j=1}^{n}W_{ij}\ln X_{ijk,t}+\mu_{it}+\lambda_{it}+\varepsilon_{it}$$

where $\theta_{k}$ means the spatial autocorrelation coefficient of the independent variables and control variables.

Besides, independent variables’ coefficients from SDM regression results do not express the marginal effects accurately [36]. Marginal effects, including direct effect and indirect effect, provide some key information for explaining the model. The direct effect indicates the influence of the local independent variables on the local dependent variables, while the indirect effect indicates the potential influence of the local independent variables on all other regional dependent variables through spatial interaction. We convert SDM into the form as follows [35]:

(6) $$Y_{it}={\left(Ι-\rho W\right)}^{-1}(X_{it}\beta +WX_{it}\theta +\mu_{it}+\lambda_{it}+\varepsilon_{it})$$

where *I* means the (*N* × 1) identity matrix, and *N* is the number of cross sections (the number of sample provinces).

The matrix of partial differential equations of the dependent variable *Y* to the *k*-th independent variable *X* is expressed as:

(7) $$\left[\frac{\partial y}{\partial X_{ik}}\cdots \frac{\partial y}{\partial X_{Nk}}\right]=\left[\begin{array}{ccc} \frac{\partial y_{1}}{\partial X_{ik}} & \cdots & \frac{\partial y_{1}}{\partial X_{Nk}}\\ \vdots & \vdots & \vdots \\ \frac{\partial y_{N}}{\partial X_{ik}} & \cdots & \frac{\partial y_{N}}{\partial X_{Nk}} \end{array}\right]={\left(Ι-\rho W\right)}^{-1}\left[\begin{array}{cccc} \beta_{k} & \omega_{12}\theta_{k} & \cdots & \omega_{1N}\theta_{k}\\ \omega_{21}\theta_{k} & \beta_{k} & \cdots & \omega_{2N}\theta_{k}\\ \vdots & \vdots & \vdots & \vdots \\ \omega_{N1}\theta_{k} & \omega_{N2}\theta_{k} & \cdots & \beta_{k} \end{array}\right]$$

In the right matrix of Equation (7), the elements in the diagonals refer to direct effects. The simple average for all the diagonal elements means the direct effect on average. Off-diagonal elements indicate the indirect effects. The simple average for all the off-diagonal elements means the indirect effect on average. The sum of the direct effect and indirect effect means the total effect on average.

We estimate all the above spatial models by applying the maximum likelihood estimation method for controlling the simultaneity caused by the introduction of equal space-weighted variables into the equation proposed by [35]. Spatial testing is performed according to the LM test method. If the LM-lag or LM-err test results are significant, the model will be estimated using SAR model or SEM spatial form. If both test results are significant, SDM should be used. We apply the spatial Hausman tests for testing whether random effects or fixed effects should be used in the estimations. In the condition of the fixed effect, we test whether time or spatial fixed effects should be included in the model by using the likelihood ratio (LR) test.

## 4. Variables and Data

### 4.1. Air Pollutant Emissions

There are no uniform indicators for comprehensively and systematically measuring the overall level of air pollutant emissions. Studies have generally used specific pollutant emission indicators to characterize air pollution [31,37]. The emission intensity of air pollution, defined as the logarithm of air pollutant emissions of a province in its unit geographical area, is used to represent the pollution intensity of a specific province. This study uses two indicators of air pollutant emissions, (a) tons of SO_2_ emissions per square kilometer and (b) tons of SD emissions per square kilometer, because they better reflect the effects of rapid industrialization and urbanization on air pollutant emissions in China over the past 20 years [38]. SO_2_ is mainly derived from mining as well as hard coal, lignite, and petroleum used in smelting activities. In contrast, SD comes from a relatively diverse industry.

### 4.2. Air Pollution Control Policy

Air pollution control policy involves both policy formulation and policy implementation [39]. At the policy formulation level, air pollution control policy refers to various atmospheric environment laws and regulations formulated and implemented by the government for environmental protection [40]. Air pollution control policy described in this paper refers specifically to all the policies and measures promulgated by the central government related to the prevention and control of air pollution.

The intensity of policy content is an indicator that describes the stringency of air pollution control policy content [20]. Albrizio et al. [39] used a new comprehensive index of Environmental Policy Stringency (EPS) developed by the Organization for Economic Co-operation and Development (OECD) in the study. The indicator ranges from 0 to 6, with higher numbers associated with more stringent environmental policies. The novelty of this indicator is that it simplifies a complex set of multidimensional strategies into comparable country-specific agents. Similar as the quantitative criteria used for EPS, we assign the value of 5 to 1 to the contents of air pollution control policies in China to describe the stringency of policy content, with recommendations of relevant legal experts and scholars (shown in Table 1). The following steps were used in the scoring: (a) train the scoring personnel and multi-group scoring by multiple groups of people to quantify the national environmental policy, (b) divide 20 policy researchers into 10 groups to score the national environmental policy in accordance with [20] scoring steps and requirements, and (c) use the homogeneity reliability method to test the quantitative data of the policy content. According to the general requirements of the Cronbach α index, when Cronbach α > 0.7, the reliability results are better. The homogeneity reliability analysis result is Cronbach α = 0.914, which indicates that the credibility of the policy content quantitative data is higher.

This paper constructs an indicator describing the stringency of air pollution control policy. $ps_{it}$ measures the stringency of the central government’s policy *i* to prevent air pollutant emissions in year *t*. The stringency of the cumulative policies is given by the sum of all $ps_{it}$:

(8) $$PS_{t}=\sum_{i=1}^{N}ps_{it}$$

Equation (8) reflects the degree to which the central government attaches importance to air pollutant emissions. The greater the air pollution control policy, the greater the importance the central government attaches to air pollutant emissions. Since all levels of government in China are governed by one party, the central government’s air pollution control policy can play an important role in all provinces across the country.

The above *PS* indicator is a measure of law and policy and does not reflect differences in policy implementation across provinces [39]. In fact, in the process of air pollution control, the central government is responsible for the formulation of air pollution control policy, and the implementation of air pollution control policy needs to be carried out at the local level [4]. Therefore, we need to include the implementation intensity of air pollution control policy at the level of provincial government. Based on the work of [41,42], we choose the proportion of provincial government environmental workers to the total number of people (*PE*), which reflects the resources used by provincial governments to manage air pollutant emissions. This paper draws on this research idea to construct provincial-level policy implementation data and uses traditional and commonly used total investment in environmental governance for robustness verification. Finally, we construct air pollution control policy intensity index by multiplying *PS* by *PE* as:

(9) $$PSPE_{it}=PS_{t}\times PE_{it}$$

### 4.3. Informal Environmental Regulation

Langpap and Shimshack [43] used citizen litigation records as proxy variables. Kathuria [7] pointed out that informal regulation can be measured by the number of articles on pollution in the domestic media. According to the informal approach to environmental regulation in China, we use two variables to describe informal environmental regulation: (a) the number of letters that the public complains about pollution and environmental related issues through official channels. This measures the public environmental participation in informal environmental regulation [3]. (b) Regional emergency environmental event data to measure media environmental monitoring. In general, areas with more environmental emergencies tend to attract more attention from the news media [3].

### 4.4. Control Variables

According to previous studies, economic development, industrial structure, S&T expenditure, energy consumption, population density, and degree of opening-up may all affect air pollutant emissions. Following existing research practices, we control them in the model to eliminate the effects of other variables.

(1) Economic development. GDP is the representative of economic growth and has a direct impact on air pollutant emissions [31]. Compared with underdeveloped areas, areas with higher economic development usually have more resources and capabilities for environmental governance. To control the impact of per capita economic scale on air pollutant emissions, we control the per capita GDP (*PGDP*) in this paper.
(2) Industrial structure. The industrial layout and industrial development scale are closely related to environmental quality. Different industrial structures correspond to different pollution discharge structures [5]. We use the industrial structure (*IND*) variable, calculated as the proportion of the secondary industry’s GDP to the total GDP, to control the impact of regional industrialization development on air pollutant emissions.
(3) S&T expenditure. Previous studies have shown that S&T has a positive impact on environmental protection [32]. Based on this, we construct the technology expenditure ratio (*TEC*) variable to control the impact of S&T expenditure on air pollutant emissions by calculating the proportion of provincial government S&T expenditure to the total fiscal expenditure of the year.
(4) Energy consumption. Energy consumption is a key factor affecting air pollutant emissions. Al-Mulali and Ozturk [33] found that energy consumption and air pollution showed a positive long-term two-way relationship. To control the impact of energy consumption on air pollutant emissions, we use the per capita energy consumption (*PEC*) for control.
(5) Population density. Population size is one of the biggest drivers of atmospheric pollutant emissions [31]. Considering the large differences in administrative divisions and population size between provinces, the direct use of absolute population indicators is not scientifically comparable. Therefore, we use population density (*PPOP*), the population per unit area, to characterize the impact of population agglomeration on air pollutant emissions.
(6) The degree of opening-up. The degree of opening-up reflected by *FDI* is an essential factor for China’s environmental pollution research. Existing research shows that the direction of FDI impact on environmental quality is not certain. For example, Pollution Halo Hypothesis suggests that FDI can improve environmental quality by introducing environmentally friendly technologies and products [34], but Pollution Haven Hypothesis argues that FDI can deteriorate its environmental quality by transferring highly polluting industries to host countries [44]. We use the proportion of FDI in GDP to measure the degree of opening-up to examine its impact on China’s air pollutant emissions.

Based on the data availability, this paper used the panel data of 30 provinces in China (excluding Hong Kong, Macao, Taiwan, and Tibet) to empirically analyze the impact of air pollution control policy and informal environmental regulation on air pollutant emissions in consideration of its spatial characteristics. The air pollutant emissions data, provincial air pollution control policy implementation data, and informal environmental regulation data were derived from China Environment Yearbooks. Air pollution control policy formulation data was obtained through policy quantification discussed above. Other variable data comes from China Statistical Yearbooks. The variables and data sources involved in this paper are shown in Table 2.

### 4.5. Descriptive Statistics

**Figure 2:**
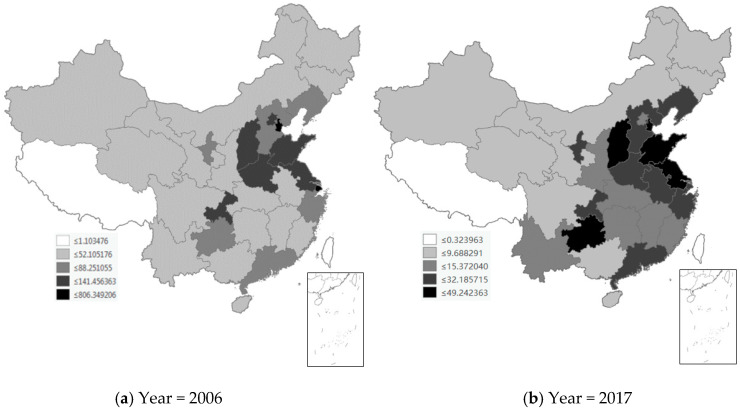
Spatial distribution of SO_2_ emission intensity in China’s 30 provinces.

**Figure 3:**
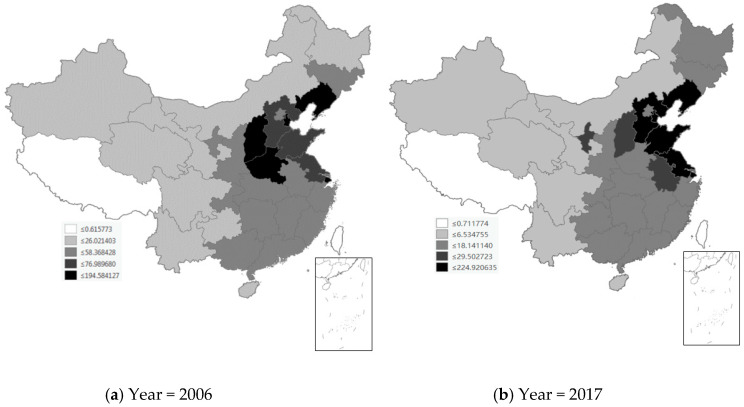
Spatial distribution of SD emission intensity in China’s 30 provinces.

**Figure A1:**
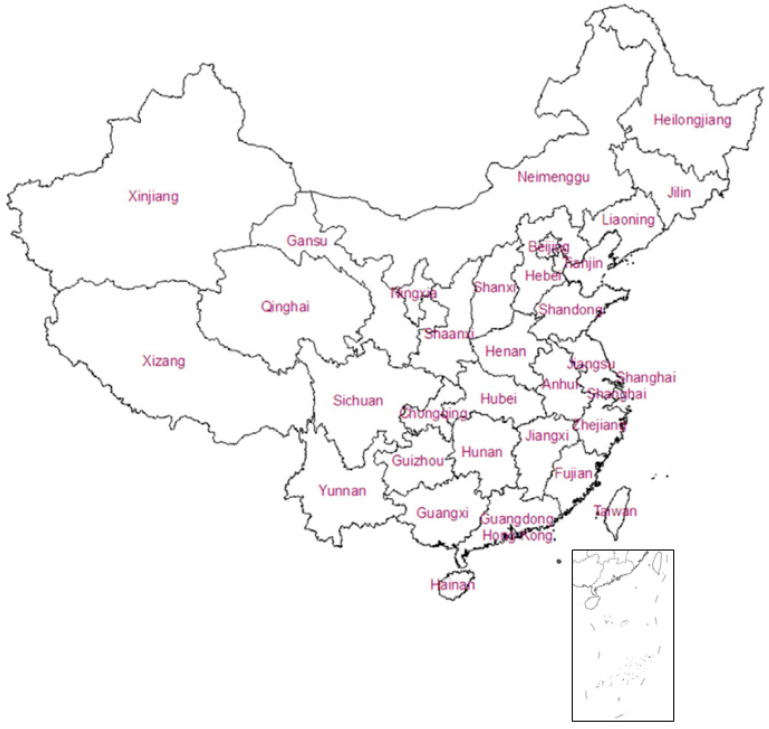
Spatial distribution of Chinese provinces [42].

We first used the Geographic Information System (GIS) to plot the spatial distribution of key dependent variables in Figure 2 and Figure 3. The spatial distribution of Chinese provinces is shown in Figure A1. Figure 2 shows the spatial distribution of SO_2_ emission intensity in China’s 30 provinces in 2006 and 2017, where the unit of SO_2_ emission intensity is thousand tons/ten thousand square kilometers. In 2006, SO_2_ emission intensity showed the characteristics of uneven spatial distribution. SO_2_ high-emission provinces were scattered in the following areas in China: Beijing, Tianjin, Hebei, Liaoning, Shandong, Jiangsu, Shanghai, Zhejiang, and Guangdong in Eastern China, Henan and Shanxi in Central China, and Chongqing, Guizhou, and Ningxia in Western China. In 2017, although such pollutants were still concentrated in the above regions, the difference in SO_2_ emission intensity between regions has been reduced and is more dispersed across the country. Figure 3 shows the spatial distribution of SD emission intensity in China’s 30 provinces in 2006 and 2017, where the unit of SD emission intensity is thousand tons/ten thousand square kilometers. In 2006, the high SD emission intensity is concentrated in these contiguous regions of China: Tianjin, Hebei, Liaoning, Shandong, Jiangsu, and Shanghai in Eastern China, and Henan and Shanxi in Central China. The map in 2017 highlights the concentration trend, making SD emission intensity more visible in these areas.

Table 3 lists the summary statistics and correlation matrices for all variables in the 30 provinces. The correlation between the dependent variables and air pollution control policy is negative, while the correlation between the dependent variables and informal environmental regulation is positive. We need to carefully interpret these pairwise correlations because they present only contemporaneous effects, and do not explain the moderating effects and spatial dependence contained in econometric analysis. Unlike the spatial regression analysis in this paper, the correlation here does not tell the directionality and time relationship between variables. The multi-collinearity is further examined by examining the correlation coefficient values between variables and calculating the variance inflation factor (VIF). All values are within an acceptable range with an average VIF of 2.80.

## 5. Results and Discussion

### 5.1. Spatial Regression Analysis

The regression results are reported from Table 4, Table 5, Table 6, Table 7, Table 8, Table 9 and Table 10 based on the panel data for 10 years. To identify potential spatial effects, it is necessary to examine the existence and form of spatial dependence before implementing spatial panel regression. We applied two LM tests and two robust LM tests to the spatial lag dependent variable and the spatial error correlation. Two LM tests and their robust forms are reported in models of Table 4 and Table 5, respectively. In Table 4, two LM tests and their robust forms are significant at the 5% significance level. This result does not reject the SEM or SAR model, which confirms that SDM can more appropriately explain the result when SO_2_ emission intensity is the dependent variable. In Table 5, the Robust LM Error is significant at the 1% significance level while the Robust LM Lag does not pass the 10% significance test, so the SEM is more explanatory for samples with SD emission intensity as the dependent variable. These results indicate that spatial models are more appropriate than traditional panel data. Therefore, the SDM in Table 4 and the SEM in Table 5 were performed in accordance with [35,36], respectively.

The spatial Hausman test is significant in all models. Therefore, we used spatial fixed effects to control the characteristics of provinces with unobserved time and space. In addition, all results of the LR tests are significant at the 1% significance level, indicating the joint significance of the spatial fixed effects in the model. Based on obtaining similar significance results, we find that the spatial fixed effect model is more reasonable through further comprehensive analysis of the R^2^ value and the economic meaning of the estimated coefficients under different fixed effects.

Due to the existence of spatial autocorrelation, the SDM regression coefficients of the independent variables cannot be expressed as a direct marginal effect, and the spatial lag coefficients of the independent variables cannot accurately reflect the space spillover effect. Based on SDM regression coefficients, we used direct, indirect, and total effects calculations to reflect the impact of air pollution control policy and informal environmental regulation on air pollutant emissions [35,36], which is shown in Table 6.

In the case where the dependent variable is the SO_2_ emission intensity, the coefficient of the spatial weight matrix W*PSO_2_ based on the geographical distance is not significant, indicating the spatial spillover effect of SO_2_ emission intensity between neighboring provinces in China is not significant although it shows a spatial negative correlation in the entire relevant model. In the case where the dependent variable is the SD emission intensity, the coefficient of the spatial weight matrix based on geographical distance is significantly positive, indicating that there is a significant spatial spillover effect on SD pollution in China’s provinces. The change of SD pollution in one province is affected not only by the SD pollution in neighboring provinces but also by the error of structural differences between regions. This structural difference is reflected in the differences among air pollution control policy, informal environmental regulation, economic development, industrial structure, S&T expenditures, energy consumption, population density, opening-up, and other spatial factors that are not included in the basic model.

It can be seen from Table 4, Table 5 and Table 6 that air pollution control policy has a significant negative effect on SO_2_ and SD emission intensity in consideration of the spatial dependence of air pollutant emissions. However, in the entire model, air pollution control policy has no significant effect on the spatial effects of SO_2_ in neighboring provinces, which will be explained in the discussion below.

In this paper, two interaction variables, air pollution control policy and public environmental participation (PSPE*PPTC) and air pollution control policy and media environmental supervision (PSPE*SEE), are introduced in model (3) and model (4) or model (6) and model (8), respectively, and are introduced together in model (5) or model (10). In the complete model (10) of Table 5, public environmental participation represented by the environmental letter has a significant inhibitory effect on SD emission intensity. The coefficient of the interaction term PSPE*PPTC is significantly negative, indicating that public environmental participation has a negative moderating effect on the negative relationship between air pollution control policy and SD emission intensity.

In Table 6 and the complete model (5) of Table 4, media environmental supervision represented by sudden environmental events has a significant inhibitory effect on SO_2_ emission intensity. The coefficient of the interaction term PSPE*SEE is significantly negative, indicating that the media environmental supervision has a negative moderating effect on the negative relationship between air pollution control policy and SO_2_ emission intensity.

### 5.2. Discussions

It is worth noting that the negative spatial spillover effect of SO_2_ emission intensity is not significant and the positive spatial spillover effect of SD emission intensity is significant, which may be related to the emission properties of these pollutants. SD is produced from a variety of sources in industrial processes in various regions, such as coal combustion, metal smelting, and processing, and cement and concrete production. Therefore, SD pollution is difficult to reduce across regions. Given the interlinkages between the cross-regional industrial value chains, increased demand from upstream or downstream sectors of a province’s value chain can increase supply in neighboring provinces, and vice versa [45,46]. As a result, industry growth and associated pollution emissions in one province can drive production and pollution levels in upstream or downstream industries in adjacent areas of the industrial supply chain. In contrast, SO_2_ comes mainly from a few sources, such as burning sulfur-intensive fuels and mining activities. Technologies that reduce SO_2_ emissions are relatively easy to implement, using “pipe end” technology to retain sulfur content or directly replacing low-sulfur fuels [47]. In addition, reducing SO_2_ emissions can also be achieved by shutting down mines or coal-fired power stations, as well as shutting down small glass, cement, and refinery plants [48]. The negative correlation between SO_2_ emissions in the provinces may reflect the concentration of such pollutants from one province to the neighboring provinces, and the growing size of such pollutants in a certain region. On the other hand, as can be seen from Figure 2, SO_2_ emissions are concentrated in large numbers between several provinces such as Beijing, Tianjin, and Hebei [31], resulting in a positive correlation between SO_2_ emissions in local provinces. This may be the main reason why the negative correlation between SO_2_ emissions in the provinces at the national level is not significant.

Air pollution control policy has a significant negative effect on SO_2_ and SD emission intensity. On one hand, air pollution control policy can directly increase the pollution costs of polluting enterprises [41], thereby promoting the reduction of air pollutant emission intensity in various regions. On the other hand, the authors of [49] show that environmental policies can effectively promote ecological innovation. Ecological innovation is a broad concept that includes pollution control, green products, clean process technologies, green energy technologies, and transportation technologies, as well as innovations in waste reduction and treatment technologies [50]. The inhibition of atmospheric pollution emissions by ecological innovation is self-evident [51], which can significantly reduce the emission intensity of SO_2_ and SD. The conclusions of this paper are consistent with the validity of the “weak” version of the Porter hypothesis [52].

Public environmental participation has a significant inhibitory effect on SD emission intensity. This result effectively complements existing research. Studies have shown that citizens can influence the location of polluting enterprises through direct environmental letters, forcing polluting enterprises to move out [3]. The relocation of polluting enterprises will obviously help reduce the SD emissions of industrial enterprises. The public environmental participation has a negative moderating effect on the negative relationship between air pollution control policy and SD emission intensity. This shows that with the increase in public environmental participation, the impact of air pollution control policy on reducing SD emission intensity has gradually increased.

Media environmental supervision has a significant inhibitory effect on SO_2_ emission intensity. This is consistent with reality. Unexpected environmental events such as the anti-molybdenum-copper project in Shifang City in 2012 eventually forced SO_2_ heavy polluting enterprises to move out of the city [3]. Moreover, the results are consistent with findings reported in [7] that if the public continues to be interested in pollution news, the news media can act as an informal regulator to inhibit corporate emissions in industrial parks. The media environmental supervision has a negative moderating effect on the negative relationship between air pollution control policy and SO_2_ emission intensity. This shows that with the strengthening of media environmental supervision, the impact of air pollution control policy on reducing SO_2_ emission intensity has gradually increased. A study by [43] shows that private law enforcement in the US encroaches on public law enforcement. The moderating effect results in this paper are inconsistent with the above conclusions, indicating that China’s informal environmental regulation (*PPTC* and *SEE*) have increased the effectiveness of air pollution control policy through different ways to pay attention to environmental violations.

In terms of control variables, per capita GDP has a significant negative effect on SD emission intensity, which may be related to the gradual growth of China’s green economy. Pretty [53] found that unlike the environmental pollution caused by traditional industrial activities, the development of the green economy will obviously reduce the concentration of environmental pollution such as particulate matter concentration. The industrial structure has a significant negative effect on SD emission intensity. This may be because the industrial restructuring of the secondary industry in China’s provinces is mainly reflected in the transformation from roughing processing to finishing processing, and from contaminated products to cleaning products, indicating a new industrialization road of coordinated development of environment and economy has achieved initial results. It can be predicted that this effect will become more apparent when the industrial structure adjustment reaches a suitable height.

The increase in the level of S&T expenditure is conducive to the reduction of SO_2_ emission intensity. SO_2_ emissions are mainly from a few heavily polluting industries and increasing the technology spending in these areas can significantly improve the pollution intensity of heavily polluting industries. This is consistent with the results of previous studies. Abdouli and Hammami [54] showed that advanced high-level technologies can reduce the level of environmental pollution. The coefficient of the impact of per capita energy consumption on SO_2_ and SD emissions is significantly positive, indicating that SO_2_ and SD emission intensity increase with the increase of per capita energy consumption. The increase in the degree of opening-up is conducive to a significant reduction in SO_2_ pollution. The research in this paper shows that the Pollution Haven Hypothesis for SO_2_ is not established in China. The degree of opening-up represented by *FDI* is likely to inhibit SO_2_ pollution through different mechanisms such as income effect, pollution halo effect [34], and technology spillover effect [29]. In addition, the spatial spillover effect of *FDI* on neighboring areas is significantly positive, indicating that a 10% increase in *FDI* in one province will result in an increase in SO_2_ emission intensity of 6.78% in neighboring provinces.

### 5.3. Robust Tests

To further assess the robustness of the regression results, two additional robust tests were performed. First, we used an alternative spatial weight matrix, the contiguity-based binary spatial weight matrix, instead of the spatial distance-based weight matrix [55] to reflect the spatial dependence of air pollutant emission intensity. From Table 7 and Table 8, we find that basic result remains the same, but there is still a slight change. The coefficient of the spatial interaction term W*PSPE*SEE has a weak significance, indicating that SO_2_ emission intensity decreases in one province due to the high-intensity air pollution control policy in the adjacent province, which is because they spatially absorb and spread the spillover effects of media environmental supervision. FDI has a weak positive effect on SD emission intensity, which provides weak evidence for the existence of the Pollution Haven Hypothesis for SD in neighboring provinces in China.

Second, there may be endogenous problems in air pollutant emissions and other factors. For example, reverse causality may create estimation problems in regional pollution studies. The higher intensity of air pollution control policy and informal environmental regulation may result in a decrease or increase in pollution intensity, but areas with less pollution or heavy pollution may also lead to the higher intensity of air pollution control policy and informal environmental regulation [5]. Therefore, we chose one period lagged for all explanatory variables as the instrumental variables of the original explanatory variables to cope with the possible causal inversion problem, substituting them into the spatial econometric models. From Table 9 and Table 10, we find that the underlying results remain the same, but there are still some minor changes. The positive spatial effect of *PPTC_1* is significant, indicating that the increase in direct public participation in a province will significantly contribute to the increase in SO_2_ emission intensity in the next time phase of the adjacent province. The coefficient of the spatial interaction term W*PSPE_1*PPTC_1 is slightly significant, indicating that the increase of SO_2_ emission intensity in the next time period of one province is due to the high-intensity air pollution control policy in the adjacent province. It is likely that they have produced a pollution crowding effect with direct public participation in time and space. In the section of control variables, the negative effect of *IND_1* on SO_2_ emission intensity becomes slightly significant, indicating that industrial structure adjustment has a significant time lag effect for SO_2_ emissions. The negative spatial spillover effect of *TEC_1* has a weak significance, indicating that the increase of S&T expenditure in one province has a time lag effect to reduce the intensity of SO_2_ emissions in neighboring provinces. The negative effect of *PGDP_1* on SD emission intensity is no longer significant, indicating that per capita GDP has no significant time lag effect for SD emissions.

## 6. Conclusions

With rapid economic growth, China’s provinces are currently facing serious air pollution problems. Effective control of air pollutant emissions has become one of the priority objectives of all levels of government in China. On the premise of considering the spatial dependence of air pollutant emissions, this paper uses the panel data of 30 provinces in China to study the impact of air pollution control policy and informal environmental regulation on air pollutant emissions, and further analyze the moderating effects of informal environmental regulation on the relationship between air pollution control policy and air pollutant emissions.

The empirical results of this paper are summarized below. First, the spatial dependence of the air pollutant emissions is not the same. The negative spatial spillover effect of SO_2_ emission intensity is not significant, while SD emission intensity has a significant positive spatial spillover effect. Second, air pollution control policy has a significant negative impact on air pollutant emission intensity. Air pollution control policy is likely to inhibit air pollutant emissions by directly increasing the pollution costs of polluting enterprises and effectively promoting ecological innovation. Third, informal environmental regulation represented by public environmental participation and media environmental supervision has significant inhibitory effects on emission intensity of air pollutants. Finally, informal environmental regulation has a negative moderating effect on the negative relationship between air pollution control policy and emission intensity of air pollutants. In addition, SO_2_ emission intensity decreases in one province due to the high-intensity air pollution control policy in the adjacent province, which is because they spatially absorb and spread the spillover effects of media environmental supervision.

Based on the findings revealed in this study, the following policy recommendations can be proposed.

First, spatial heterogeneity plays an important role in environmental regulation in China. Local governments should fully realize that if they do not cooperate with other neighboring regions, it is impossible to continuously improve the air pollution control in each province, and fundamentally solve the problem of air pollutant emissions such as acid rain and haze. Economically developed provinces cannot reduce air pollutant emissions simply by transferring highly polluting industries to neighboring provinces. While such interventions may have a temporary impact, they will eventually increase emissions of air pollutants in neighboring provinces and endanger local air quality.

Second, policy formulation and policy implementation are very important for air pollution prevention and control. Air pollution control policy described in this paper can reflect the policy formulation of the central government and the policy implementation of local governments. Chinese central government should formulate new air pollution control policy for specific air pollution issues based on adhering to existing air pollution control policies. At the same time, China’s provincial governments should continue to strengthen policy implementation. In addition, based on the results of the robustness test, local governments should fully recognize the persistence of the air pollution governance process and maintain the coherence of specific local environmental protection measures in the process of implementing air pollution control policy.

Third, science and technology are the most important means for controlling air pollutant emissions. There is a strong need to properly direct FDI to invest in technologies related to air pollution control. Governments and businesses need to strengthen S&T spending in the field of cleaner production. Local governments need to focus on monitoring technology application that is indeed being adopted by businesses in various regions.

Finally, the important role played by informal environmental regulation in controlling air pollutant emissions should be strengthened. The public citizen should raise awareness of environmental protection further and participate in environmental supervision voluntarily. Public environmental participation should be encouraged and promptly responded by the governments. Moreover, traditional media and online media should increase the supervision of the process of air environmental governance and report on illegal sewage companies legally. Governments should give more support to various media for legal environmental protection supervision.

There are some limitations to this study. First, air pollution control policy includes different policy instruments such as command-and-control instruments and market-oriented instruments [4]. We did not address the different policy instruments of air pollution control policy due to the focus of this study. Second, we measured the overall level of air pollutant emissions as the dependent variables, without considering the complicated structures of air pollutant emission sources such as the motorized traffic, which may have affected the lower moderating effects in this study. The research can be further explored from these aspects in the future.

## Figures and Tables

**Table 1:** Quantitative standards for the stringency of air pollution control policy contents.

Scores	Quantitative Criterion	
5	Make sure the legal status or enforced requirements for reducing and preventing the atmospheric pollutant emissions; formulate mandatory standards for reducing the atmospheric pollutant emissions; forcibly require strictly implementing environmental impact assessment, formulating prevention of pollution program and implementing “three simultaneousness” system; enforce to implement charging discharge fees system, establish new credit or price punitive system for pollution projects, require eliminating equipment of high pollution and high emissions; require formulating relevant policies to promote air pollution prevention and control from the legislation; formulate enforced methods to promote preventing air pollution, etc.	Detailed
3	Clearly require reducing atmospheric pollutant emissions, formulate specific embodiment of air pollution prevention; support pollution prevention and control from the aspects of administrative licensing, taxation, finance and fees, and also formulate support program; forcibly require strictly implementing environmental impact assessment, formulating prevention of pollution program and implementing “three simultaneousness” system; have formulated pollutant recycling program and program for eliminating equipment of high pollution and high emissions; have formulated a clear air pollution prevention and control goals, but not required to enforce, etc.	General
1	Only mention the above terms without formulated relevant measures and methods.	Mentioned

**Table 2:** Variable definition table.

Type	Variable	Variable Name	Data Source
Air pollutant emissions	*PSO_2_*	SO_2_ emission intensity	China Environment Yearbook Website: (https://navi.cnki.net/KNavi/YearbookDetail?pcode=CYFD&pykm=YHJSD&bh=)
	*PSD*	SD emission intensity	
Air pollution control policy	*PSPE*	Air pollution control policy intensity	Policy quantification [20] and China Environment Yearbook Website: (https://navi.cnki.net/KNavi/YearbookDetail?pcode=CYFD&pykm=YHJSD&bh=)
Informal environmental regulation	*PPTC*	Public environmental participation	China Environment Yearbook Website: (https://navi.cnki.net/KNavi/YearbookDetail?pcode=CYFD&pykm=YHJSD&bh=)
	*SEE*	Media environmental monitoring	
Control variables	*PGDP*	Per capita GDP	China Statistical Yearbook Website: (http://www.stats.gov.cn/tjsj/ndsj/)
	*IND*	Industrial structure	
	*TEC*	Technology expenditure ratio	
	*PEC*	Per capita energy consumption	
	*PPOP*	Population density	
	*FDI*	Degree of opening-up	

**Table 3:** Descriptive statistics and correlation matrix.

Variables	Mean	SD	1	2	3	4	5	6	7	8	9	10	11
1. *PSO_2_*#	3.458	1.139	1.000										
2. *PSD*#	3.074	1.005	0.925 ***	1.000									
3. *PSPE*#	12.620	0.505	−0.239 ***	−0.123 **	1.000								
4. *PPTC*#	7.037	3.438	0.368 ***	0.269 ***	−0.579 ***	1.000							
5. *SEE*#	2.167	1.186	0.373 ***	0.317 ***	−0.205 ***	0.123 **	1.000						
6. *PGDP*#	5.660	0.562	0.264 ***	0.305 ***	0.447 ***	−0.280 ***	0.161 ***	1.000					
7. *IND*	0.464	0.081	0.177 ***	0.172 ***	−0.060	0.372 ***	−0.055	−0.176 ***	1.000				
8. *TEC*	0.019	0.013	0.486 ***	0.468 ***	−0.135 **	0.006	0.415 ***	0.660 ***	−0.284 ***	1.000			
9. *PEC*#	3.519	0.540	0.001	−0.006	0.471 ***	−0.284 ***	−0.044	0.584 ***	0.042	0.289 ***	1.000		
10. *PPOP*#	5.441	1.278	0.818 ***	0.805 ***	−0.147 ***	0.100 *	0.397 ***	0.450 ***	−0.159 ***	0.668 ***	−0.127 **	1.000	
11. *FDI*	0.387	0.520	0.200 ***	0.195 ***	−0.141 ***	−0.024	0.244 ***	0.279 ***	−0.340 ***	0.413 ***	0.033	0.406 ***	1.000

Note: # indicates the natural logarithm for variables. *, **, and *** indicate statistically significant at the levels of 10%, 5%, and 1%, respectively.

**Table 4:** Regression results of spatial fixed effect spatial Durbin model (SDM) for SO_2_ emission intensity.

DV: *PSO_2_*	Model (1)	Model (2)	Model (3)	Model (4)	Model (5)
*PGDP*	0.061 (0.369)	0.088 (0.315)	0.049 (0.300)	0.014 (0.277)	0.015 (0.262)
*IND*	−0.338 (0.300)	−0.368 (0.269)	−0.293 (0.266)	−0.332 (0.218)	−0.289 (0.219)
*TEC*	−0.043 ** (0.018)	−0.044 ** (0.017)	−0.041 ** (0.017)	−0.034 ** (0.015)	−0.031 ** (0.014)
*PEC*	0.772 *** (0.127)	0.790 *** (0.129)	0.781 *** (0.125)	0.838 *** (0.144)	0.834 *** (0.141)
*PPOP*	−0.034 (0.539)	−0.235 (0.422)	−0.079 (0.465)	−0.080 (0.407)	0.108 (0.444)
*FDI*	−0.051 *** (0.015)	−0.048 *** (0.013)	−0.046*** (0.013)	−0.041 *** (0.013)	−0.039 *** (0.013)
*PSPE*		−0.257 *** (0.088)	−0.247 ** (0.097)	−0.217 ** (0.089)	−0.200 ** (0.096)
*PPTC*			−0.007 (0.007)		−0.012 (0.008)
PSPE*PPTC			−0.036 (0.029)		−0.037 (0.025)
*SEE*				−0.013 * (0.007)	−0.014 * (0.007)
PSPE*SEE				−0.044 ** (0.02)	−0.046 ** (0.020)
W*PGDP	−0.884 (0.572)	−0.647 (0.58)	−0.550 (0.799)	−0.899 (0.559)	−0.767 (0.800)
W*IND	0.494 (0.566)	0.671 (0.529)	0.831 (0.726)	0.804 * (0.444)	0.698 (0.655)
W*TEC	−0.204 *** (0.056)	−0.197 *** (0.063)	−0.196 *** (0.08)	−0.155 ** (0.064)	−0.130 (0.091)
W*PEC	−0.029 (0.580)	−0.124 (0.553)	−0.381 (0.999)	0.172 (0.515)	0.125 (0.962)
W*PPOP	2.808 (2.394)	2.620 (2.212)	2.149 (2.265)	1.628 (2.113)	0.786 (2.355)
W*FDI	0.598 *** (0.148)	0.626 *** (0.15)	0.532 *** (0.154)	0.732 *** (0.135)	0.678 *** (0.170)
W*PSPE		0.091 (0.172)	0.209 (0.340)	0.221 (0.191)	0.162 (0.275)
W*PPTC			−0.006 (0.023)		0.013 (0.023)
W*PSPE*PPTC			0.015 (0.088)		0.048 (0.085)
W*SEE				−0.001 (0.025)	0.002 (0.027)
W*PSPE * SEE				−0.135 * (0.078)	−0.132 (0.092)
W*PSO2	−0.045 (0.309)	−0.056 (0.297)	−0.055 (0.275)	−0.139 (0.304)	−0.063 (0.279)
LM-LAG	3.610 **	4.672 ***	4.657 **	3.661 *	2.988 *
Robust LM-LAG	16.852 ***	18.968 ***	16.605 ***	19.271 ***	16.658 ***
LM-ERR	328.001 ***	389.331 ***	356.630 ***	374.515 ***	321.366 ***
Robust LM-ERR	341.243 ***	403.627 ***	368.578 ***	390.125 ***	335.036 ***
LR test spatial effect	668.250 ***	674.030 ***	709.260 ***	690.380 ***	707.940 ***
Spatial Hausman tests	19.350 ***	149.260 ***	147.790 ***	14.120 **	20.090 **
R^2^	0.600	0.618	0.633	0.648	0.663

Note: The robust standard error clustering at the provincial level is given in the brackets; *, **, and *** indicate statistically significant at the levels of 10%, 5%, and 1%, respectively.

**Table 5:** Regression results of spatial fixed effect spatial error model (SEM) for SD emission intensity.

DV: *PSD*	Model (6)	Model (7)	Model (8)	Model (9)	Model (10)
*PGDP*	−0.787 ** (0.316)	−0.493 ** (0.245)	−0.595 ** (0.234)	−0.486 ** (0.251)	−0.595 ** (0.239)
*IND*	−1.056 *** (0.307)	−1.227 *** (0.293)	−1.135 *** (0.294)	−1.233 *** (0.295)	−1.137 *** (0.295)
*TEC*	−0.020 (0.027)	−0.019 (0.023)	−0.017 (0.022)	−0.022 (0.024)	−0.019 (0.023)
*PEC*	0.747 *** (0.151)	0.807 *** (0.163)	0.799 *** (0.148)	0.796 *** (0.163)	0.796 *** (0.149)
*PPOP*	0.477 (0.888)	0.333 (0.603)	0.324 (0.684)	0.290 (0.609)	0.303 (0.681)
*FDI*	0.001 (0.022)	0.006 (0.017)	0.011 (0.016)	0.009 (0.018)	0.014 (0.017)
*PSPE*		−0.393 *** (0.132)	−0.345 *** (0.131)	−0.391 *** (0.143)	−0.341 ** (0.14)
*PPTC*			−0.039 *** (0.015)		−0.038 ** (0.015)
PSPE*PPTC			−0.061 * (0.033)		−00.061 * (0.033)
*SEE*				0.008 (0.01)	0.005 (0.011)
PSPE*SEE				−0.002 (0.026)	−0.006 (0.023)
Spatial effects W	0.734 *** (0.086)	0.727 *** (0.066)	0.720 *** (0.070)	0.725 *** (0.066)	0.719 *** (0.071)
LM-LAG	78.765 ***	68.331 ***	67.412 ***	59.220 ***	55.780 ***
Robust LM-LAG	0.216	0.868	0.779	1.569	1.844
LM-ERR	389.975 ***	314.106 ***	317.177 ***	256.673 ***	236.446 ***
Robust LM-ERR	311.426 ***	246.642 ***	250.545 ***	199.022 ***	182.51 ***
LR test spatial effect	475.05 ***	469.51 ***	492.53 ***	463.04 ***	479.58 ***
Spatial Hausman tests	68.680 ***	18.680 ***	498.730 ***	16.560 **	110.670 ***
R^2^	0.321	0.349	0.386	0.353	0.389

Note: The robust standard error clustering at the provincial level is given in the brackets; *, **, and *** indicate statistically significant at the levels of 10%, 5%, and 1%, respectively.

**Table 6:** Direct, indirect, and total effects of SDM in model (5).

DV: *PSO_2_*	Direct Impact	Indirect Impact	Total Impact
*PGDP*	0.032 (0.270)	−0.648 (0.758)	−0.616 (0.714)
*IND*	−0.286 (0.231)	0.754 (0.695)	0.468 (0.696)
*TEC*	−0.030 * (0.015)	−0.128 (0.094)	−0.158 * (0.093)
*PEC*	0.848 *** (0.143)	−0.063 (0.970)	0.786 (0.935)
*PPOP*	0.120 (0.448)	0.885 (2.511)	1.005 (2.261)
*FDI*	−0.040 *** (0.013)	0.675 ** (0.285)	0.635 ** (0.292)
*PSPE*	−0.198 ** (0.099)	0.160 (0.299)	−0.038 (0.31)
*PPTC*	−0.013 (0.008)	0.012 (0.024)	0.000 (0.026)
PSPE*PPTC	−0.035 (0.025)	0.049 (0.089)	0.013 (0.079)
*SEE*	−0.014 * (0.007)	0.004 (0.027)	−0.011 (0.028)
PSPE*SEE	−0.047 ** (0.019)	−0.130 (0.12)	−0.177 (0.123)

Note: The robust standard error clustering at the provincial level is given in the brackets; *, **, and *** indicate statistically significant at the levels of 10%, 5%, and 1%, respectively.

**Table 7:** Regression results of the spatial fixed effect SDM for SO_2_ emission intensity under the contiguity-based binary spatial weight matrix.

DV: *PSO_2_*	Model (A1)	Model (A2)	Model (A3)	Model (A4)	Model (A5)
*PGDP*	−0.144 (0.341)	−0.127 (0.312)	−0.152 (0.288)	−0.171 (0.278)	−0.201 (0.261)
*IND*	−0.349 (0.284)	−0.379 (0.257)	−0.231 (0.230)	−0.329 (0.228)	−0.183 (0.185)
*TEC*	−0.043 ** (0.018)	−0.041 ** (0.017)	−0.040 ** (0.018)	−0.032 * (0.018)	−0.032 * (0.018)
*PEC*	0.695 *** (0.191)	0.741 *** (0.196)	0.701 *** (0.192)	0.803 *** (0.201)	0.766 *** (0.194)
*PPOP*	0.210 (0.819)	−0.014 (0.810)	0.450 (0.852)	0.141 (0.730)	0.624 (0.758)
*FDI*	−0.071 *** (0.022)	−0.067 *** (0.021)	−0.075 *** (0.021)	−0.061 *** (0.023)	−0.069 *** (0.022)
*PSPE*		−0.255 *** (0.092)	−0.214 ** (0.100)	−0.198 ** (0.098)	−0.158 * (0.098)
*PPTC*			−0.005 (0.009)		−0.008 (0.010)
PSPE*PPTC			−0.048 (0.033)		−0.049 (0.031)
*SEE*				−0.013 * (0.007)	−0.013 * (0.007)
PSPE*SEE				−0.042 * (0.023)	−0.039 ** (0.019)
LW*PGDP	−0.533 (0.400)	−0.302 (0.399)	−0.181 (0.426)	−0.460 (0.392)	−0.326 (0.417)
LW*IND	0.383 (0.614)	0.507 (0.586)	0.492 (0.604)	0.497 (0.505)	0.480 (0.516)
LW*TEC	−0.029 (0.045)	−0.03 (0.048)	−0.020 (0.040)	−0.024 (0.044)	−0.014 (0.039)
LW*PEC	−0.028 (0.386)	−0.087 (0.397)	−0.167 (0.419)	0.028 (0.375)	−0.056 (0.392)
LW*PPOP	−0.783 (1.466)	−0.423 (1.444)	−1.022 (1.440)	−0.573 (1.296)	−1.252 (1.278)
LW*FDI	0.188 ** (0.078)	0.196 *** (0.074)	0.229 *** (0.074)	0.213 *** (0.075)	0.250 *** (0.067)
LW*PSPE		−0.008 (0.189)	−0.036 (0.199)	0.006 (0.205)	−0.017 (0.197)
LW*PPTC			−0.007 (0.009)		−0.003 (0.010)
LW*PSPE*PPTC			0.047 (0.050)		0.057 (0.042)
LW*SEE				−0.001 (0.013)	−0.003 (0.014)
LW*PSPE*SEE				−0.041 (0.039)	−0.054 * (0.029)
LW*PSO_2_	0.128 (0.163)	0.099 (0.155)	0.164 (0.167)	0.064 (0.150)	0.137 (0.156)
Spatial Hausman tests	44.960 ***	30.330 ***	15.830 **	1099.780 ***	19.950 **
R^2^	0.548	0.570	0.599	0.600	0.631

Note: The robust standard error clustering at the provincial level is given in the brackets; *, **, and *** indicate statistically significant at the levels of 10%, 5%, and 1%, respectively.

**Table 8:** Regression results of the spatial fixed effect SEM for SD emission intensity under the contiguity-based binary spatial weight matrix.

DV: *PSD*	Model (A6)	Model (A7)	Model (A8)	Model (A9)	Model (A10)
*PGDP*	−0.798 *** (0.186)	−0.492 ** (0.199)	−0.596 *** (0.189)	−0.469 ** (0.207)	−0.574 *** (0.196)
*IND*	−1.074 *** (0.314)	−1.253 *** (0.292)	−1.120 *** (0.302)	−1.273 *** (0.292)	−1.134 *** (0.303)
*TEC*	−0.020 (0.026)	−0.014 (0.023)	−0.016 (0.022)	−0.018 (0.023)	−0.020 (0.023)
*PEC*	0.658 *** (0.157)	0.742 *** (0.174)	0.697 *** (0.154)	0.724 *** (0.172)	0.683 *** (0.154)
*PPOP*	0.262 (0.804)	0.073 (0.565)	0.234 (0.664)	0.015 (0.571)	0.197 (0.665)
*FDI*	0.007 (0.014)	0.014 (0.012)	0.019 * (0.011)	0.016 (0.012)	0.021 * (0.012)
*PSPE*		−0.388 *** (0.14)	−0.325 ** (0.142)	−0.396 *** (0.151)	−0.333 ** (0.152)
*PPTC*			−0.035 ** (0.014)		−0.034 ** (0.014)
PSPE*PPTC			−0.069 ** (0.031)		−0.068 ** (0.031)
*SEE*				0.011 (0.011)	0.009 (0.011)
PSPE*SEE				0.011 (0.028)	0.009 (0.024)
Spatial effects LW	0.476 *** (0.085)	0.482 *** (0.077)	0.473 *** (0.079)	0.482 *** (0.079)	0.475 *** (0.083)
Spatial Hausman tests	13.890 **	17.520 **	44.790 ***	157.110 ***	41.310 ***
R^2^	0.344	0.360	0.403	0.361	0.403

Note: The robust standard error clustering at the provincial level is given in the brackets; *, **, and *** indicate statistically significant at the levels of 10%, 5%, and 1%, respectively.

**Table 9:** Regression results of the spatial fixed effect SDM for SO_2_ emission intensity based on the explanatory variables lagged 1-time phase.

DV: *PSO_2_*	Model (B1)	Model (B2)	Model (B3)	Model (B4)	Model (B5)
*PGDP_1*	−0.003 (0.353)	0.010 (0.323)	0.075 (0.322)	−0.040 (0.295)	0.039 (0.302)
*IND_1*	−0.360 (0.276)	−0.384 (0.253)	−0.414 (0.255)	−0.361 (0.225)	−0.405 * (0.213)
*TEC_1*	−0.062 *** (0.017)	−0.063 *** (0.016)	−0.065 *** (0.014)	−0.057 *** (0.015)	−0.059 *** (0.012)
*PEC_1*	0.673 *** (0.116)	0.686 *** (0.118)	0.684 *** (0.116)	0.719 *** (0.131)	0.718 *** (0.131)
*PPOP_1*	−0.249 (0.524)	−0.413 (0.460)	−0.273 (0.477)	−0.320 (0.459)	−0.141 (0.465)
*FDI_1*	−0.066 *** (0.014)	−0.064 *** (0.012)	−0.056 *** (0.014)	−0.059 *** (0.011)	−0.045 *** (0.013)
*PSPE_1*		−0.196 ** (0.085)	−0.195 ** (0.089)	−0.164 * (0.088)	−0.159 * (0.092)
*PPTC_1*			−0.002 (0.005)		−0.005 (0.005)
PSPE_1*PPTC_1			−0.011 (0.022)		−0.011 (0.018)
*SEE_1*				−0.005 * (0.009)	−0.006 * (0.009)
PSPE_1*SEE_1				−0.037 * (0.023)	−0.041 ** (0.021)
W*PGDP_1	−0.254 (0.438)	−0.111 (0.497)	−0.046 (0.594)	−0.210 (0.485)	−0.099 (0.634)
W*IND_1	0.992 ** (0.491)	1.118 ** (0.476)	0.477 (0.615)	1.199 *** (0.401)	0.369 (0.562)
W*TEC_1	−0.200 *** (0.064)	−0.201 *** (0.071)	−0.191 *** (0.073)	−0.163 ** (0.079)	−0.131 * (0.074)
W*PEC_1	−0.589 (0.393)	−0.661 (0.41)	−0.213 (0.856)	−0.511 (0.422)	0.119 (0.819)
W*PPOP_1	3.710 (2.437)	3.621 (2.215)	2.770 (2.395)	2.917(2.212)	1.346 (2.572)
W*FDI_1	0.650 *** (0.187)	0.667 *** (0.194)	0.698 *** (0.179)	0.726 *** (0.192)	0.862 *** (0.189)
W*PSPE_1		0.113 (0.252)	−0.293 (0.399)	0.181 (0.244)	−0.305 (0.315)
W*PPTC_1			0.026 (0.021)		0.042 ** (0.019)
W*PSPE_1*PPTC_1			0.113 (0.075)		0.168 * (0.097)
W*SEE_1				−0.009 (0.028)	0.018 (0.041)
W*PSPE_1*SEE_1				−0.078 (0.086)	−0.133 (0.096)
W*PSO_2_	0.227 (0.192)	0.220 (0.191)	0.108 (0.211)	0.242 (0.195)	0.129 (0.198)
Spatial Hausman tests	21.270 ***	25.440 ***	19.790 **	80.970 ***	131.880 ***
R^2^	0.595	0.606	0.621	0.620	0.646

Note: The robust standard error clustering at the provincial level is given in the brackets; *, **, and *** indicate statistically significant at the levels of 10%, 5%, and 1%, respectively.

**Table 10:** Regression results of the spatial fixed effect SEM for SD emission intensity based on the explanatory variables lagged 1-time phase.

DV: *PSD*	Model (B6)	Model (B7)	Model (B8)	Model (B9)	Model (B10)
*PGDP_1*	−0.403 (0.267)	−0.115 (0.216)	−0.195 (0.207)	−0.131 (0.231)	−0.220 (0.223)
*IND_1*	−0.872 *** (0.313)	−1.044 *** (0.315)	−1.004 *** (0.299)	−1.047 *** (0.311)	−1.003 *** (0.299)
*TEC_1*	−0.004 (0.025)	−0.003 (0.024)	−0.002 (0.023)	−0.006 (0.024)	−0.005 (0.023)
*PEC_1*	0.438 *** (0.131)	0.497 *** (0.14)	0.499 *** (0.131)	0.500 *** (0.147)	0.509 *** (0.138)
*PPOP_1*	0.298 (0.884)	0.160 (0.647)	0.060 (0.76)	0.116 (0.656)	0.029 (0.747)
*FDI_1*	−0.033 (0.022)	−0.028 (0.017)	−0.024 (0.016)	−0.021 (0.015)	−0.018 (0.015)
*PSPE_1*		−0.383 *** (0.141)	−0.347 ** (0.138)	−0.363 ** (0.148)	−0.323 ** (0.144)
*PPTC_1*			−0.033 ** (0.016)		−0.033 ** (0.015)
PSPE_1*PPTC_1			−0.035 * (0.034)		−0.034 * (0.032)
*SEE_1*				0.010 (0.012)	0.007 (0.012)
PSPE_1*SEE_1				−0.028 (0.031)	−0.032 (0.029)
Spatial effects W	0.731 *** (0.066)	0.710 *** (0.067)	0.693 *** (0.07)	0.703 *** (0.07)	0.688 *** (0.072)
Spatial Hausman tests	10.900 **	12.140 **	16.660 **	16.060 **	27.530 ***
R^2^	0.158	0.228	0.268	0.244	0.281

Note: The robust standard error clustering at the provincial level is given in the brackets; *, **, and *** indicate statistically significant at the levels of 10%, 5%, and 1%, respectively.
